# Low-carbohydrate diet in type 2 diabetes. Stable improvement of bodyweight and glycemic control during 22 months follow-up

**DOI:** 10.1186/1743-7075-3-22

**Published:** 2006-06-14

**Authors:** Jørgen Vesti Nielsen, Eva Joensson

**Affiliations:** 1 Department of Medicine, Blekingesjukhuset, Karlshamn. 37480 Karlshamn, Sweden

## Abstract

**Background:**

Low-carbohydrate diets in the management of obese patients with type 2 diabetes seem intuitively attractive due to their potent antihyperglycemic effect.

We previously reported that a 20 % carbohydrate diet was significantly superior to a 55–60 % carbohydrate diet with regard to bodyweight and glycemic control in 2 non-randomised groups of obese diabetes patients observed closely over 6 months. The effect beyond 6 months of reduced carbohydrate has not been previously reported. The objective of the present study, therefore, was to determine to what degree the changes among the 16 patients in the low-carbohydrate diet group at 6-months were preserved or changed 22 months after start, even without close follow-up. In addition, we report that, after the 6 month observation period, two thirds of the patients in the high-carbohydrate changed their diet. This group also showed improvement in bodyweight and glycemic control.

**Method:**

Retrospective follow-up of previously studied subjects on a low carbohydrate diet.

**Results:**

The mean bodyweight at the start of the initial study was 100.6 ± 14.7 kg. At six months it was 89.2 ± 14.3 kg. From 6 to 22 months, mean bodyweight had increased by 2.7 ± 4.2 kg to an average of 92.0 ± 14.0 kg. Seven of the 16 patients (44%) retained the same bodyweight from 6 to 22 months or reduced it further; all but one had lower weight at 22 months than at the beginning. Initial mean HbA1c was 8.0 ± 1.5 %. After 6 and 12 months it was 6.6 ± 1.0 % and 7.0 ± 1.3 %, respectively. At 22 months, it was still 6.9 ± 1.1 %.

**Conclusion:**

Advice on a 20 % carbohydrate diet with some caloric restriction to obese patients with type 2 diabetes has lasting effect on bodyweight and glycemic control.

## Background

We previously reported that 16 obese patients with type 2 diabetes, who were advised to lower their carbohydrate intake to 20 %, over 6 months achieved significantly better control of hyperglycemia and bodyweight than a control group of similar patients (n = 15), advised to follow the official dietary guidelines where 55–60 % carbohydrate is recommended [1]. We have now reviewed the clinical charts after 22 months and present the data for the patients. Since two-thirds of the controls at some point have changed diet, we report here mainly the results for the 20 %-carbohydrate group with regard to glycemic control (HbA1c), bodyweight, body mass index (BMI) (kg/m^2^) and lipids after 22 months. We also report HbA1c and bodyweight for the 7 patients who immediately switched to a 20 % carbohydrate diet from the original low-fat diet, and for whom long-term data is available

## Methods

The method has previously been described in detail [1]. In short, the patients – free of thyroid illness, manifest cardiac and renal disease – were advised to follow a diet containing initially 1800 kcal for men and 1600 kcal for women. The proportions of carbohydrates, protein and fat were 20 %, 30 % and 50 % respectively. The daily quantity of carbohydrates was 80–90 g. The recommended carbohydrate consumption was limited to vegetables and salad. Instead of ordinary bread crisp/hard bread was recommended, each slice containing 3.5 to 8 g carbohydrates. Excluded were bread, pasta, potatoes, rice and breakfast cereals. The patients were counselled to not eat between meals. It was further recommended that they walk 30 minutes a day and take a daily multivitamin supplement containing extra calcium. There was an introductory meeting lasting most of a day. The subjects were then followed closely for 6 months.

The controls were initially advised on a diet with about the same caloric content, but the proportions of carbohydrates, protein and fat were 55–60 %, 15 % and 25–30 % respectively [1]. In the normal diabetes diet whole-grain products are recommended. Generous helpings of vegetables and several servings of fruits as snacks between meals are also recommended. As a number of the controls attended our normal diabetes educational course as introduction to the observation period, the control group on average received about 50 % more attention – measured in hours – than the low-carbohydrate group. The controls then proceeded in the same way as the low-carbohydrate group.

Seven of the controls switched to a 20 % carbohydrate diet immediately after the follow-up period. For those we have data 12–14 months after the change. Three more have later sought information and have changed diet. We have no long-term data for those. Five of the original controls have not changed diet.

All the patients were known to us and visited the diabetes nurse regularly after the initial period. The same scales and laboratory were used for all measurements. The present report is a review of clinical charts. Where a figure is missing at 22 months we have taken the mean from the two closest figures. Means are given with standard deviations. T-test for dependent samples is used.

## Results

Table 1 shows the measured parameters from start to 22 months.

**Table 1:** Effect of diet on weight, BMI, HbA1c and fasting lipids. Sixteen obese patients with type 2 diabetes started at month 0 on a diet with the proportions: 20 % carbohydrates, 30 % protein and 50 % fat. The figures shown are before, 3, 6 and 22 months after the dietary change

**Months**	**0**	**3**	P*	**6**	P*	**22**	P*
**Weight **(kg)	**100.6 **± 14.7	**91.9 **± 14.7	<0.001	**89.2 **± 14.3	<0.001	**92.0 **± 14.0	<0.001
**BMI **(kg/m^2^)	**36.1 **± 4.2	**33.0 **± 4.5	<0.001	**32.0 **± 4.3	<0.001	**32.9 **± 3.5	<0.001
**HbA1c **(%)	**8.0 **± 1.5	**5.9 **± 0.7	<0.001	**6.6 **± 1.0	<0.001	**6.9 **± 1.1	<0.001
**Lipids**(mmol/l)							
**Tot-Chol**.	**5.6 **± 1.2	**5.8 **± 1.1	0.4	**6.1 **± 1.1	0.06	**5.7 **± 1.2	0.9
**HDL-Chol.**	**1.1 **± 0.2	**1.2 **± 0.2	<0.002	**1.3 **± 0.2	<0.001	**1.3 **± 0.3	<0.001
§ **Triglycerides**	**1.4**(1;1,8)	**1.2**(0.8;1,4	0.01	**1.4**(0.9;1.7)	0.4	**1.4**(1.2;1.9)	0.9
**Chol/HDL**	**5.4 **± 1.5	**5.0 **± 1.5	0.02	**5.0 **± 1.7	0.07	**4.6 **± 1.6	<0.001
§ **TG/HDL**	**1.4**(0.9;1.7)	**1.0**(0.6;1.2)	0.003	**1.0**(0.7;1.5)	0.03	**1.3**(0.8;1.5)	0.2

§ given as median with 25 and 75 percentiles. * p values are for differences from baseline.

### Bodyweight

**Figure 1:**
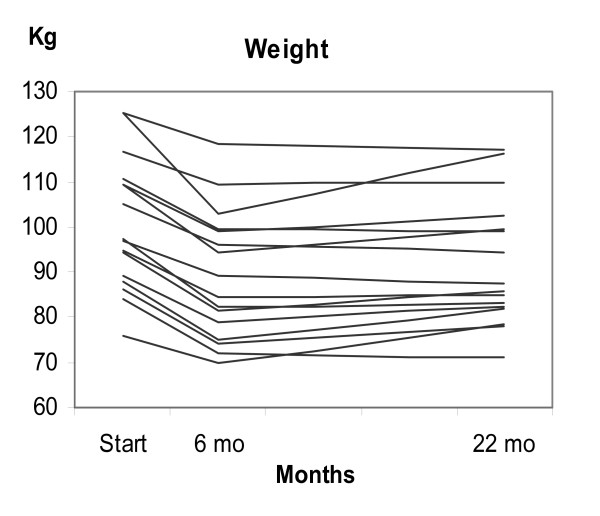
**Individual changes in bodyweight in 16 obese patients with type 2 diabetes**. The patients at start changed from a high-carbohydrate diet to a diet consisting of 20 % carbohydrates, 30 % protein and 50 % fat.

The mean weight has increased from month 6 to month 22 by 2.7 ± 4.2 kg. Seven of the 16 patients retained the same bodyweight from 6 to 22 months or reduced it further (See Figure 1).

### HbA1c

Initial mean HbA1c for the low carbohydrate group was 8.0 ± 1.5 %. After 6 months the value was 6.6 ± 1.0 % and, after 12 months, 7.0 ± 1.3 % (normal HbA1c < 5.6 % in non-diabetic persons).

The mean HbA1c for the group has been stable since the 12 month mark. At 22 months mean HbA1c was 6.9 ± 1.1 % and 4 patients displayed an HbA1c below 6.0 % whereas initially only one person did so.

**Figure 2:**
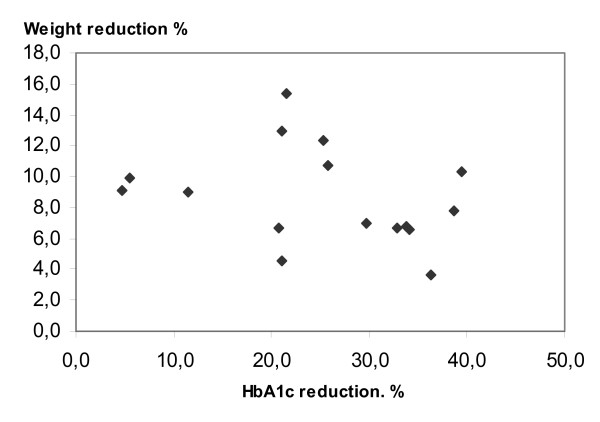
**Percentage changes in HbA1c and bodyweight in 16 obese patients with type 2 diabetes**. The figures are plotted 3 months after a change to a 20 % carbohydrate diet at which time the reduction in HbA1c was most pronounced.

The HbA1c reduction occurred independently of the weight reduction as seen in Figure 2 where the relationship between percentage reduction of weight and HbA1c after 3 months is shown (r = -0.30).

### Medications

An important feature of carbohydrate restriction as a treatment for type 2 diabetes is that reduction or elimination of medication is often possible [8,21,22]. At the start of this study, 15 of the 16 patients used metformin and 5 sulfonylurea (SU). Eleven patients were insulin-treated. The mean daily insulin dosage was 60 ± 33 IU/day.

At the end of the 6-months 20 %-carbohydrate period 2 persons had discontinued SU and 3 had reduced the doses. Three subjects had discontinued insulin and the mean insulin requirement among the last 8 persons had fallen to 18 ± 11 IU/day.

Over the next 16 months 2 persons have again started insulin treatment following an increased intake of carbohydrates. The mean requirement of the 11 persons is 27 ± 21 IU/day.

Fourteen subjects used antihypertensive medications. Five reduced the medications during the first 6 months and have not increased them again. The mean blood pressure has been unchanged. Six of the patients used statins. There has been no change.

### The cross-over of the 7 controls

The 7 controls were followed for 2 periods of 6 months separated by a gap of 2 months. As shown in Table 2, reduction of bodyweight and HbA1c in the low-fat period was 3.5 ± 3.5 kg and 0.9 ± 0.8 % respectively. Both bodyweight and HbA1c increased slightly in the 2 month gap period but the change to a low-carbohydrate diet led to a mean reduction of bodyweight during the 20 %-carbohydrate period of 7.5 ± 6.4 kg and of HbA1c of 0.9 ± 1.1 % (range: 0–2.4). The weight and HbA1c achieved during the 20% carbohydrate period were retained over the following 6 months (see Table 2).

**Table 2:** Changes for the low fat group during cross-over to low carbohydrate

**Intervention**	**Month**	**Weight change **(kg)	**HbA1c %**
**Low-fat**	**1–6**	**-3.5 **± 3.5 (range: 0 to -9)	**-0.9 **± 0.8 (range: -0.4 to -1.8
**Gap**	**7–8**	**0.5 **± 1.6	**0.3 **± 0.3
**Low CHO**	**9–14**	**-7.5 **± 6.4 (range: 0 to -20)	**-0.9 **± 1.1 (range: 0 to -2.4)
		**Mean Weight **(kg)	**Mean HbA1c **(%)
**Low-CHO**	**15**	**89.2 **± 15.9	**5.7 **± 0.8
	**22**	**90.2 **± 15.5	**5.7 **± 0.9

§given as median with 25 and 75 percentiles. * p values are for differences from baseline.

For the five controls, who never changed diet, there was no average change over the 18–22 months.

## Discussion

The short-term effectiveness of low-carbohydrate diets for weight reduction is well established [2-6]. Weight reduction is primarily caused by decreased caloric intake [2-6] although decreased energy efficiency has also been found [7]. A high-starch, high-carbohydrate diet excessively stimulates appetite and disturbs energy balance in patients with the metabolic syndrome and type 2 diabetes [8]. A reduction of carbohydrates normalises the balance, reduces insulin concentrations and favours utilization of stored fat as fuel as well as significantly reducing insulin resistance [8]. Weight loss in overweight persons is improved by a higher proportion of protein, presumably due to protein's effect on satiety and/or metabolic efficiency [7,9-11]. A reduction in carbohydrates for patients with type 2 diabetes effectively reduces both fasting and postprandial glucose as well as HbA1c. These effects can be independent of weight loss [8,12,13].

Critics of the low-carbohydrate dietary approach usually point to the lack of long-term studies. The change of diet described here in combination with the usual diabetes care has produced long periods of increased well-being in these patients. It is significant that 44 % of the patients have had a stable weight or have reduced it further and all but one had a lower weight at 22 months than at the beginning of the study. Twenty-five % of the patients had previously been dieting continuously and another 50 % had tried to lose weight many times. The reasons they gave for not continuing their previous weight loss programs were 1) constant hunger, and 2) no effect on bodyweight despite strict calorie counting and fat reduction. The lack of hunger and cravings with the low-carbohydrate diet may be an important reason for their present success.

Regular self measurement of blood glucose has probably made it possible for these patients to succeed. Self determination of blood glucose provides a feed-back mechanism and may be another reason that 44 % of the patients succeeded in maintaining their weight.

For most insulin-treated patients, we found that it was necessary to be one step ahead in lowering the insulin doses, that is, if weight loss stopped, and the patient was adhering to the diet (as judged by blood glucose values), even a minor reduction of the insulin dose normally resulted in continued weight loss and presumably continued motivation. This close supervision may be another reason for the patients' initial success.

Improved HbA1c is associated with a reduced incidence of microvascular and macrovascular complications [14-16]. The original low carbohydrate group had maintained a significantly lower mean HbA1c for almost 2 years and a reduced vascular morbidity might therefore be expected in this group. Similarly, intentional weight loss in type 2 diabetes patients is associated with a reduced mortality of 30–40 % [17]. For the average patient each 1 kg weight loss is associated with 3–4 months prolonged survival [18] making it likely that the patients described here have achieved a survival benefit.

At some point following a reduction of bodyweight and insulin resistance, a decrease of cardiovascular risk would be expected [19,20]. We have examined the medical charts for both the original high-carbohydrate group and the low-carbohydrate group from 3 months after the initiation of the diet therapy – when an effect might be detected – and forward for episodes of cardiovascular disease. Three episodes of cardiovascular disease have occurred among the 5 patients that never changed diet. The 16 patients in the low-carbohydrate diet group (19 months observation time) and the 7 from the high-carbohydrate diet group that changed diet (10 months observation time) – totalling 23 patients – have been free of cardiovascular disease during the follow-up period (p < 0.03. Fischer Exact).

Several recent reviews have made the case for reducing the carbohydrate load in type 2 diabetes [21-23] or metabolic syndrome [24] and the low-carbohydrate diet presented here is clearly effective in many obese people with type 2 diabetes. Because of its effectiveness it should be used with close clinical supervision in patients on insulin or oral hypoglycaemic agents.

In summary, a reduced carbohydrate diet is an effective tool in the management in motivated obese patients with type 2 diabetes. The effect is generally retained after almost 2 years. There has been no evidence of a negative cardiovascular effect among the 16 subjects.

## Competing interests

The author(s) declare that they have no competing interests.

## Authors' contributions

JVN wrote the manuscript and analysed the data. Both authors collected data and gave final approval to the manuscript.
